# CHLA 2026 CONFERENCE CONTRIBUTED PAPERS / ABSC CONGRÈS 2026 COMMUNICATIONS LIBRES

**DOI:** 10.29173/jchla29968

**Published:** 2026-08-03

Sophie Fillion, Cristina Leblanc

CISSS de la Montérégie-Est

Face à l’évolution rapide des besoins cliniques, à l’émergence fulgurante de l’intelligence artificielle dans les milieux de la santé et de la recherche, à la pression croissante sur les espaces cliniques, ainsi qu’à l’arrivée de la société d’état Santé- Québec, la Bibliothèque du Centre intégré de santé et de services sociaux de la Montérégie-Est (CISSSME) a saisi l’occasion de se réinventer à travers un projet de transformation innovant. Souhaitant offrir un vent de fraîcheur aux services de la bibliothèque, en plus de pallier à une légère diminution des activités traditionnelles — notamment la recherche documentaire et le prêt de documents — l’équipe de la Bibliothèque a élaboré ce projet à la suite d’une revue de la littérature. Le projet s’articule autour de cinq volets : la mise en place d’une résidence de bibliothécaire directement au sein des services cliniques, le déménagement et la centralisation de deux succursales, la veille et l’accompagnement en intelligence artificielle, le développement des services aux chercheurs, étudiants et gestionnaires, ainsi que l’ouverture des services aux patients. Inspirés par des initiatives menées au Québec, ailleurs au Canada et à l’international, ces différents volets progressent à des rythmes variés, chacun comportant ses propres défis, mais générant déjà des résultats prometteurs. Cette conférence offrira une vue d’ensemble des services de la bibliothèque et de son évolution dans le milieu, ainsi qu’une vue en profondeur du volet « résidence », intitulé « Loin des murs, près de vous ». La présentation mettra en lumière les avancements réalisés au cours de la dernière année et proposera des pistes concrètes pour les bibliothèques souhaitant actualiser leur offre de services.

