# Supplementary material for: CHLA 2026 CONFERENCE CONTRIBUTED PAPERS / ABSC CONGRÈS 2026 COMMUNICATIONS LIBRES
Source: J Can Health Libr Assoc. 2026 Aug 3;47(2):57–96. doi: 10.29173/jchla29968 (PMC13590149; doi:10.29173/jchla29968)
Supplement: Related texts

# Abstract: CP2. Expert-recommended tasks for hospital librarians during a healthcare system merger or acquisition: an e-Delphi consensus statement

Stacy Posillico^1^, Jaclyn Morales^1^, Saori Wendy Herman^2^

^1^Northwell Health, ^2^Zucker School of Medicine at Hofstra Northwell

**Introduction:** Little empirical research exists to guide hospital librarians through healthcare system mergers or acquisitions. To address this gap, an e-Delphi research study was used to develop expert-driven recommendations prioritizing those tasks librarians should undertake when consolidating library services in newly merged, geographically distributed healthcare systems. **Methods:** A literature review through January 2021, updated October 2023, identified relevant publications but no research. Twenty-nine hospital librarians who had merger experience in the last ten years within the United States participated as experts in a modified e-Delphi study conducted according to CREDES standards. The panel completed four questionnaire rounds from April to December 2022. In Round 1, they identified tasks librarians should undertake during system mergers. In Rounds 2 to 4, tasks rated 5, 6, or 7 on a Likert Scale by ≥75% of panelists were included in the final consensus statement. **Results:** A statement of 330 recommended tasks for librarians to consider during healthcare system mergers and acquisitions was created. Recommended tasks are organized within four domains: Healthcare Organization, Library Collections & Information Systems, Library Administration, and Library Staff Integration & Interconnection. Tasks related to information technology services, vendor relations, and library organizational structure were more likely to be prioritized than tasks involving marketing and physical space. Expert insight highlighted understanding context and culture when undertaking recommended tasks. **Discussion:** This research introduces consensus-based actionable tasks to consider in future mergers and acquisitions. Collaboration with Canadian hospital librarians to adapt and validate the recommendations within provincial governance and funding contexts is invited.

# Abstract: CP3. Health innovation, entrepreneurship, and commercialization: a dialogue in shaping future health libraries supports and services

Jeff Mason

McMaster University

**Introduction:** A growing body of literature demonstrates a role for libraries to support innovation, entrepreneurship, and commercialization activities at their institutions and in the broader start-up community. Despite multi-trillion-dollar global health care markets, there are few examples of how medical libraries can help advance organizational priorities focused on translating research and clinical innovations into viable health care products and services. This presentation aims to explore the landscape of medical library support for innovation, entrepreneurship, and commercialization and begin a conversation about how medical library workers can collaborate with students, faculty, staff, and other affiliated users they support to become an integral part of the innovation community. **Description:** Using examples from the literature and the authors' experiences, this presentation introduces how libraries help advance organizational and community goals to support and grow innovation, entrepreneurship, and commercialization initiatives, with a focus on health care. The information needs and information seeking behaviours of entrepreneurs, and how these may differ from typical medical library users, will also be discussed. **Discussion:** There is an emerging opportunity for medical libraries to demonstrate their value as partners within health innovation, entrepreneurship, and commercialization ecosystems. Success may mean rethinking or reimagining the vision medical libraries have of themselves as research, learning, and clinical partners. As library budgets continue to tighten, supporting health innovation, entrepreneurship, and commercialization activities may also open the door to new funding opportunities for libraries, library workers, and the communities they support.

# Abstract: CP4. An innovative library-clinician collaboration for teaching shared decision making (SDM) in an academic institution

Natalia Tukhareli

Canadian Memorial Chiropractic College

**Introduction:** Health sciences libraries increasingly contribute to teaching evidence-based practice (EBP), with instruction typically focusing on the “Ask,” “Acquire,” and “Appraise” steps of the EBP cycle. This presentation describes an academic library’s collaboration with clinicians to teach the “Apply” step within the framework of Shared Decision Making (SDM) to undergraduate students. **Methods:** A literature review identified current trends and best practices in teaching SDM in medical education. Additional insights on student needs and curriculum capacity were obtained through consultations with faculty and clinicians. The analysis informed the selection of key SDM teaching points and effective strategies for incorporating them into an online module and the undergraduate curriculum. **Results:** Librarians and clinicians co-created six instructional videos filmed in the library and clinical settings. The recordings introduced SDM principles, components, models, barriers, and demonstrated developing and communicating evidence-based management plans. Clinician-patient role-play illustrated two contrasting cases involving similar clinical presentations but different psychosocial and socioeconomic contexts. New instructional content was also added to the library’s EBP LibGuide. **Discussion:** Since library-based EBP instruction often omits the “Apply” step, this initiative demonstrates how librarians can partner with clinicians to address it effectively. By integrating SDM principles, the project emphasized empathy and compassion as essential components of evidence-based healthcare. The initiative received positive feedback from academic and clinical departments, enhancing the library’s visibility and reputation. A formal evaluation of the library’s instruction will be conducted in the future as part of a comprehensive, program-wide assessment of EBP instruction across all four undergraduate years.

# Abstract: CP5. Competencies as a compass: exploring how academic and health sciences librarians gain foundational skills

Rachel Whitney^1^, Valerie Vera^2^, Molly K. Maloney^3^, Jessica Diaz^1^

^1^Medical University of South Carolina, ^2^University of South Carolina, ^3^University of Buffalo

**Introduction:** This study aims to examine the divide between curriculum and practice in library and information science (LIS) programs by identifying how academic and health sciences librarians acquire the foundational skills necessary for success in the field. **Methods:** An anonymous electronic survey was disseminated to practicing academic and health sciences librarians in the United States through various professional listservs. The survey captured demographic information, whether foundational skills were taught in LIS programs, if foundational skills were transferrable to practice, and how these skills were further developed after graduation. **Results:** Final results from this study are pending completion of data analysis. Primary outcomes of this study include how academic and health sciences librarians gain foundational skills, such as literature searching, efficient database navigation, effective research interview methodologies, and citation management practices. Secondary outcomes include a comparison of whether skills taught in LIS programs have changed based on graduation year and a review of current course descriptions of American Library Association (ALA)-accredited programs. **Discussion:** Reference and User Services, along with Research and Evidence-Based Practice, are key ALA competencies for the success of academic and health sciences librarians. Findings may inform iSchools and LIS educators on potential areas to enhance curriculums with these foundational skills. Additionally, the results may inform professional development opportunities for early career librarians (e.g., professional organizations), better preparing LIS students to meet the demands of the field and contribute meaningfully to the advancement of librarianship.

# Abstract: CP6. From lit review to poster: an educational intervention for RNs and APPs

Elaina Vitale^1^, Bridget Linehan^2^

^1^Dartmouth Medical and Health Sciences Libraries, ^2^Dartmouth Health

**Introduction:** A health sciences librarian and an APRN have partnered to develop a four month educational series for registered nurses (RNs) and advanced practice providers (APPs) at our institution who are interested in research, but aren't sure how to get started. **Methods/Description:** We are using a pre / post survey to gauge RN and APP confidence with creating a research question, performing a literature search, reading and understanding literature, and creating and presenting a research poster before and after a four month seminar series on these topics. **Results/Outcomes:** RN and APP research output at our institution is currently low, whether because of time constraints, institutional support, or confidence to pursue a research trajectory. Participants in our series have been guided through poster submission at a conference relevant to their clinical specialty or research interest, and in January 2026, the series will culminate in a casual poster symposium where all participants will present their research posters in front of peers and colleagues. By the time of CHLA, we will be prepared to discuss early successes of the project, pre and post survey details, and enrollment details. **Discussion:** The series itself is a model for other librarians who are interested in increasing their outreach with nursing staff or nursing students. At CHLA, we will discuss the series and series content in more detail, and share preliminary findings from the pre and post survey, as well as details of successful poster submissions from our participants.

# Abstract: CP7. Development of a knowledge synthesis searching competencies evaluation test

Amy Bergeron, Tara Landry, Monique Clar

Université de Montréal

**Context:** The Bibliothèque de santé, Université de Montréal launched a knowledge synthesis support program in the 2024-2025 academic year. In line with our goal to build skills among our current staff, we developed a tool to assess the current competency levels of our health science librarians, to offer targeted professional development recommendations. The tool was deployed in May 2025. **Methods:** The tool consisted of a questionnaire developed using Microsoft Forms online. The questionnaire was divided into two sections, each starting with a scenario in which respondents were asked to revise the search methods of a project. In the first section, participants could choose one of four project titles. They were then asked to provide feedback on three categories of the project: sources searched, the search strategy used, and reporting. An additional field for comments that did not fit into these categories was also available. A second project was proposed in the second section to complement the first. Each strategy for review was based on a published systematic review, representing clinical medicine, nursing, rehabilitation, pharmacology, and public health. MEDLINE was the primary database used in the first section, and CINAHL as the primary database used in the second. We developed a scoring system to evaluate each question according to completeness of identified issues in the three categories. Minor issues (one point) and important issues (three points) were identified for each project. **Discussion:** This presentation will address the development of the tool and the scoring system, as well as deployment and reflections on future directions.

# Abstract: CP8. Portrait des services de soutien aux synthèses des connaissances (SdC) dans les universités, les hôpitaux et les milieux spécialisés québécois

Monique Clar^1^, Bénédicte Nauche^2^, Daniela Ziegler^3^

^1^Université de Montréal, ^2^Institut de recherche Robert-Sauvé en santé et en sécurité du travail, ^3^Centre hospitalier de l'Université de Montréal

**Contexte:** Les milieux documentaires (universitaires, hospitaliers et spécialisés) québécois ont développé, dans les dernières années, une offre de service pour soutenir les revues systématiques, les examens de la portée et autres synthèses des connaissances (SdC). Les équipes, les niveaux de service, les enjeux et les solutions varient d’un milieu à l’autre. **Objectif :** Faire le portrait québécois des services de SdC. **Méthode :** Après une revue de la littérature, un questionnaire a été créé pour collecter des données notamment sur la mise en place du service, les niveaux de service, les outils, les rôles, les responsabilités et les enjeux pour l'avenir. Pour faire un portrait des services, 74 milieux (bibliothèques, services de documentation ou unités) ont été invités à répondre au questionnaire. Les répondants ont eu entre le 4 septembre et le 3 octobre 2025 pour compléter le questionnaire. **Résultats:** 43 questionnaires ont été complétés. Tous les milieux sont bien représentés. 37% (n=16) ont un service formel en place (avec des balises, par exemple : politique, procédurier, etc.). 30% (n=13) ont un service informel en place. Une institution est en voie d'offrir ce type de service et 4 sont en réflexion. 21% (n=9) des répondants n'envisagent pas d'offrir le service. Cette présentation proposera une analyse complète des résultats.

# Abstract: CP9. Quality in question: how systematic are systematic reviews in health and science disciplines?

Megan Bresnahan, Eugenia Opuda

University of New Hampshire

**Introduction:** Rapid growth in the publication of systematic reviews over the past several years has amplified the importance of librarian support and participation in evidence synthesis. Beyond serving as search strategy experts, librarians guide researchers in selecting the appropriate type of review for their research objectives and educate them about best practices and reporting guidelines. **Methods:** Using the Web of Science database, the authors identified a sample of 100 systematic reviews published in the last five years across ten health and science disciplines, including Nursing, Nutrition & Dietetics, Health Care Sciences & Services, Public Health, and Veterinary Sciences. The authors assessed the quality of the systematic reviews’ methodologies using common recommendations from best practice guidelines, such as the Cochrane Handbook and the JBI Manual for Evidence Synthesis. In each discipline, we identified trends in methodologies and reporting practices, and analyzed their correlation with journal impact factors and citations rates. **Analysis and Discussion:** Results of our analysis emphasize the variability in systematic review methodology and reporting quality. Authors frequently select inappropriate review typologies and inconsistently apply systematic review guidelines across disciplines. By understanding systematic review practices and publication trends across health and science research communities, librarians can engage more effectively with the researchers they support. By identifying common mistakes in reporting practices, librarians can raise researcher awareness about issues in syntheses rigor and publication cultures.

# Abstract: CP10. Using design thinking to shape the future of an academic library's support for knowledge synthesis

Jeff Mason, Katie Merriman, Neera Bhatnagar, Nicole Doro, Susanna Galbraith, Ines Perkovic

McMaster University Libraries

**Introduction:** An interdisciplinary working group (WG) of librarians at McMaster University Libraries in Hamilton, Ontario was charged with exploring how library support for knowledge synthesis (KS) could be refined to meet the changing needs of library users in health sciences and other research disciplines. **Description:** The WG applied a modified design thinking approach to its task. Design thinking is an iterative problem-solving approach, centred on deeply understanding user needs. It is particularly well suited for complex concerns without clear causes or solutions. This presentation explores how the five stages of design thinking — empathize, define, ideate, prototype, and test — can be applied to refining academic library support for KS. **Outcomes:** The WG developed a draft problem statement, workplan, and timeline in February 2025. The problem statement was further validated and refined after completing an environmental scan, literature review, interviews with library leadership, and focus groups with librarians and library assistants supporting KS. Solutions were ideated and 13 high impact short- and medium-term recommendations were created, validated, and refined. Next steps include prioritizing the recommendations, developing and testing solutions, and implementing and evaluating the solutions with the most promise. Results from the prototype and test stages of the project will be discussed as available. **Discussion:** Lessons learned (e.g., respecting librarian autonomy) from the application of the design thinking process and future directions for KS support at the libraries will be discussed. The authors will also reflect on how design thinking principles can be applied to other library services and contexts.

# Abstract: CP11. Shaping the future of health science libraries: RoRA as a model for navigating research currents and reclaiming library agency

Brie Marie McConnell

University of Waterloo

**Background:** Academic health sciences libraries increasingly function as active research partners, yet institutional recognition remains tied narrowly to publishable outputs. Librarians’ intellectual contributions to systematic reviews and evidence synthesis projects are frequently unacknowledged, despite substantial expertise and time investment. Moreover, research engagement across academic institutions typically unfolds in fragmented, ad-hoc patterns that prevent libraries from demonstrating measurable impact and reclaiming their narrative in the research enterprise. **Purpose:** The Records of Research Activities (RoRA) model was developed at the University of Waterloo to reconceptualize the library’s role as an intentional steward and agent of information flow. RoRA positions librarians as essential contributors whose expertise shapes research quality throughout the entire project lifecycle—not just at publication. By generating clean, consistent research activity data across five distinct stages of engagement, RoRA enables libraries to demonstrate impact, document professional contributions, and support researcher career development at multiple points beyond final publication. **Description:** RoRA employs a river metaphor to frame research as a dynamic and continually flowing process. Librarians serve as expert navigators and guides through complex information currents, exercising agency and professional judgment across five stages: Discovery and Planning (Reading the Waters), Evidence Enablement (Navigating Rapids), Creating and Analyzing (Branching Out), Dissemination (Watershed), and Reflection and Review. The model leverages a SharePoint-based research data registry indexed with MeSH terms to document consultation types, database utilization, search methodologies, and diverse research outputs. Critical to RoRA’s innovation is its reconceptualization of success metrics: research records, protocols, trial registrations, data management plans, and knowledge syntheses represent meaningful research contributions and professional achievements—independent of formal publication timelines. **Conclusion:** By generating robust research activity records while maintaining ethical boundaries and institutional privacy, RoRA transforms libraries from reactive service providers into proactive agents of research integrity and innovation. The model demonstrates that research impact extends far beyond publications, enabling libraries to own their data, control their narrative, document their intellectual contributions, and reclaim their rightful position as indispensable partners in advancing research excellence.

# Abstract: CP12. Administration and librarian perceptions of return on investment (ROI) for association leadership

Tara Landry^1^, Kristine M. Alpi^2^

^1^Université de Montréal, ^2^Icahn School of Medicine at Mount Sinai

**Context:** This research explores impact, value and return on investment (ROI) of serving in high-level leadership roles within health sciences library (HSL) professional associations as perceived by the librarian leaders and the administrators to whom they report. The objective of the first phase of the study discussed here is to gather and share insights from a global range of library leaders on the value of such service to them as individuals and their institutions. **Methods:** The librarian survey targeted two audiences. First, individuals who served in leadership roles within MLA, CHLA/ABSC, EAHIL, AHLIA, and HLA over the past ten years. Second, current library directors in the Association of Academic Health Sciences Libraries Directory of U.S. and Canadian institutions. The 27-question survey asked about motivations and perceived benefits at key points in the leadership trajectory: when participants were invited to serve, after agreeing to run for office, and during their term. The online survey included closed and open-ended questions to capture a range of experiences. Two independent analysts coded the open-ended responses, compared codes to establish a codebook, and then re-coded the data. We examined the codes to come to consensus on the themes and identify exemplar quotes. **Discussion:** From the literature and our own experiences, we anticipated themes related to professional growth, institutional visibility, and strategic alignment. Preliminary findings will be shared to foster dialogue on the value of leadership contributions within the HSL profession and to inform those considering future service.

**CP13. Apologies, apologies: Indigenous-led frameworks for librarians and medical journals to redress anti-Indigenous racism**

Warner Adam^1^, Terri Aldred^2^, Donna Atkinson^3^, Danièle Behn Smith^4^, Michael Bird^5^, Sarah de Leeuw^6^, Patricia Farrugia^7^, Elaine Kilabuk^8^, Aluki Kotierk^9^, April MacNaughton^10^, Melanie Morningstar^11^, Deanna Nyce^12^, Chantelle Richmond^13^, Jaris Swidrovich^14^, Stanley Vollant^15,^

^1^Lake Babine Nation, Chief Executive Officer, Carrier Sekani Family Services, Prince George, BC; ^2^Tl'Azt'En Nation, Academic Lead, NCCIH; Executive Medical Director of Primary Care, First Nations Health Authority; ^3^Research and Operations Manager, National Collaborating Centre for Indigenous Health; Métis, ^4^Deputy PHO – Indigenous Health, BC Office of the Provincial Health Officer; ^5^Santo Domingo-San Juan Pueblo Indian, Past President, American Public Health Association, Albuquerque, NM, USA; ^6^Director, National Collaborating Centre for Indigenous Health; ^7^Anishinaabe, Assistant Clinical Professor, Surgery, Faculty of Health Sciences, McMaster University; ^8^Nunavut Inuk, Physician, Ottawa General Hospital and Qikiqtani General Hospital; ^9^Inuk, President, Nunavut Tunngavik Incorporated; ^10^Ktunaxa Nation, Acting Director of Public Health Operations, ISC; Office of the CMO of Public Health, HSS, Indigenous Services Canada, and Adjunct Professor, UBC School of Nursing; ^11^Garden River First Nation, Acting Director of Health, Assembly of First Nations (AFN); ^12^Nisga’a First Nation, President and Chief Executive Officer of Wilp Wilxo’oskwhl Nisga’a Institute; ^13^Biigtigong Nishnaabeg, Professor and Canada Research Chair in Indigenous Health and the Environment, University of Western Ontario; ^14^Yellow Quill First Nation, Assistant Professor, Indigenous Engagement Lead, Leslie Dan Faculty of Pharmacy, University of Toronto; ^15^Pessamit First Nation, Director of Public Health, First Nations of Quebec and Labrador, Health and Social Services Commission, Wendake, Quebec

**Context:** Medical and health journals are increasingly releasing public apologies for their roles in producing and perpetuating racism. Our presentation describes our collaboration with a medical journal to identify instances of anti- Indigenous racism within its publications. This work forms part of their broader truth and reconciliation initiative, which includes publishing our findings to show how colonialism shaped publication practices related to Indigenous health. By helping develop search parameters to collate instances of anti-Indigenous racism, librarians play a vital role in current apology work. Collaborations with First Nations, Inuit, and Métis leaders are essential to this redress work. However, requests for Indigenous leaders’ assistance are often: 1) framed by longstanding rejections of Indigenous submissions to these journals; 2) anchored in fundamental misunderstandings about reconciliation and partnering with Indigenous experts; and 3) undertaken within parameters misaligned with realities of Indigenous communities. Our paper proposes strategies to overcome these barriers. **Methods/Methodologies:** This review project centres voices and reflections of First Nations, Inuit, and Métis leaders to establish anti-colonial theoretical frameworks that drive storytelling methods to understand how medical and health journals can work with First Nations, Inuit, and Métis experts. The outcomes are short vignettes about Indigenous lived experiences working with medical and health journals to combat anti-Indigenous racism. **Results/Discussion:** Actively following the directions of Indigenous leaders is crucial in producing journal content aimed at ameliorating health inequities stemming from colonial supremacy and anti-Indigenous racism. This paper offers a rare opportunity to listen closely to Indigenous voice and vision about ways to support apology work.

# Abstract: CP14. Characterizing curation: creating a readily-accessible changelog of MEDLINE indexing

Emma Garlock^1^, Alexandre Amar-Zifkin^2^

^1^University of Ottawa, ^2^Université de Montréal

**Introduction:** Anecdotes about questionable MEDLINE indexing circulate among health/medical librarians, and the National Library of Medicine works to curate them. On the basis of external librarian feedback, ‘red flags’ no longer automatically results in the MeSH ‘Emblems and Insignia’; ‘sex assigned at birth’ no longer invokes ‘Infant’. “As of April 2022, all journals indexed for MEDLINE are done by automated indexing, with human review and curation of results as appropriate.” We seek to illuminate the character of this curation, as details are challenging to access. A record’s indexing can change from one day to the next, with no indication of changes other than the Modification Date-[LR] field. Search retrieval may be impacted by reindexing; the status quo of obscurity leaves users in the dark. **Method:** Using the NLM e- utilities API, we downloaded every record added to PubMed over five days (n = 25,439), and continue to re-download them regularly. Scripts were implemented in R to identify records that changed method-of-indexing and isolate any MeSH added or removed. We summarize and describe this data. Findings 1194 records changed from automated to curated (1,084; with MeSH changes in 23%) or automated to human (110; with MeSH changes in 100%). **Discussion:** We present the world’s first public PubMed indexing changelog. This work is fundamental to gauging the impacts of changes-to- indexing on search results; these changes constitute data points for future research. Reindexing is metadata errata; greater accessibility and transparency empower users to engage more fully with literature platforms.

# Abstract: CP15. The elephant in the room: does Undermind outperform real-world knowledge synthesis search? A case study in exercise science

Erica Nekolaichuk

University of Toronto

**Introduction:** As the production of knowledge synthesis research accelerates in the field of exercise science, so has the proliferation of generative AI (genAI) academic search engines that claim to speed up the process of information retrieval —an often time-consuming and resource-intensive slog that requires specialized skills. These tools are potentially an attractive option for researchers looking to save time and resources, but it remains uncertain whether they can match the rigor of traditional search methods. Undermind is a genAI academic search engine that aims to help users “research with unprecedented breadth and depth” and uses iterative semantic search technology to search faster than a human without compromising thoroughness. This study asks: Does Undermind match or improve upon the real-world search practices reported in exercise science knowledge synthesis studies? **Methods:** This study calculated the precision and recall of the academic AI search engine Undermind against a random sample of search methods reported in 50 systematic reviews published in exercise science journals between 2015-2020. Each review question was searched the same way twice in Undermind to assess replicability. An analysis was conducted to explore patterns in the performance of Undermind. Analysis is ongoing and results will be added

# Abstract: CP16. To sum up: an overview of AI text summarization tools for literature reviews

Gary S. Atwood

University of Vermont

**Introduction:** The exponential growth of research literature is creating a massive information overload, making the traditional literature review increasingly challenging for researchers, clinicians, faculty, and students. Generative AI summarization tools offer a promising way to manage this information overload by condensing complex information into concise, accessible summaries. However, the wide range of available tools and the complexity of their underlying technology can make it difficult to assess their reliability and determine appropriate uses. **Description:** This session provides a practical overview of AI summarization tools for health information professionals. Participants will learn how these tools function and what content they can summarize (e.g. reports, full-text articles). The presentation will also highlight the different ways that summarization capabilities are offered, either through standalone tools or as integrated features in other information resources. Examples will illustrate applications across various stages of the literature review process, such as identifying themes, comparing findings, and generating overviews of search results. The session will also highlight the different ways that summarization capabilities are offered, either through standalone tools or as integrated features in other information resources. Examples will illustrate applications across various stages of the literature review process, such as identifying themes, comparing findings, and generating overviews of search results. The session will also address key limitations, such as accuracy, bias, and over-simplification, and offer guidance on responsible evaluation and use. **Outcomes:** Attendees will gain a foundational understanding of generative AI summarization technologies and their potential role in researching and writing literature reviews. They will also learn practical strategies for evaluating, recommending, and integrating these tools into instruction/reference services and research consultations.

# Abstract: CP17. Navigating institutional AI policies: educational tools for students, clinicians, and researchers

Amy Bergeron^1^, Melissa Helwig^2^, Tara Landry^1^

^1^Université de Montréal, ^2^Toronto Metropolitan University

**Introduction:** As artificial intelligence (AI) becomes increasingly integrated into health education, research, and clinical practice, institutions are releasing guidelines and policies that impact how faculty, staff, and learners engage with these technologies. In this rapidly changing landscape, current and future healthcare practitioners must quickly familiarize themselves with these guidelines and policies to adapt and shape their use of AI. To support the development of AI literacy, this project aims to create and share learning materials that facilitate understanding and application of institutional AI policies. **Description:** The team reviewed AI-related guidelines from member institutions of the Association of Faculties of Medicine of Canada (AFMC), using ChatGPT to extract common elements and build an educational framework. Policies from select healthcare institutions were also analyzed, and additional themes were incorporated. In total, twelve key themes emerged. For each theme, case-based learning scenarios were developed for diverse audiences, including students, clinicians, and researchers. Scenarios included details on population, context, use type, tool license level, and discussion prompts. Feedback from clinicians and educators helped ensure relevance and applicability. Scenarios were translated into French, and both versions are openly available. **Discussion:** Health sciences librarians are uniquely positioned to collaborate with faculty and learners in developing AI competencies and promoting best practices in information use. By leveraging open educational methods, this resource is designed to be adaptable across institutions, supporting engaging and policy-informed AI education for health and medical learners, faculty and staff.

# Abstract: CP18. A contemporary competency framework for librarians in evidence synthesis

Megan Kennedy

University of Alberta Library, Geoffrey & Robyn Sperber Health Sciences Library

Health sciences librarians are essential collaborators in the design and delivery of evidence syntheses. Earlier competency frameworks were developed with systematic reviews as the primary focus; however, evidence synthesis in the health sciences now includes scoping, rapid, realist, integrative, and mixed-method reviews. At the same time, the growing use of specialized technologies—such as AI-assisted search tools, automated screening platforms, and workflow management systems—has introduced new areas of required expertise. This project updates the competency framework from Townsend et al. (2017) for health sciences librarians engaged in evidence synthesis. Using a narrative literature review approach, we synthesized research, guidance documents, and practice-based reports describing librarian roles and activities across review types. The analysis identifies competencies from the original framework that remain essential, including database expertise, rigorous search strategy development, and leadership in promoting transparency and reproducibility. It also introduces expanded competencies reflecting current practice, such as methodological literacy across diverse review approaches; instructional and mentorship responsibilities for learners and research teams; and the strategic and ethical use of technologies supporting searching, screening, data management, and dissemination. The updated framework offers a contemporary, flexible set of competencies to guide professional development, inform curriculum design, and support institutional recognition of librarian expertise in evidence synthesis.

# Abstract: CP19. Assessing librarian involvement and impact in knowledge synthesis publications at UPEI

Keri McCaffrey, John L. Bayhi, Kim Mears, Spencer Merz-Wood

University of Prince Edward Island

**Introduction:** The University of Prince Edward Island (UPEI) formally introduced knowledge synthesis (KS) services in 2021. The main aims of this study were to determine usage of the KS service, involvement of librarians in these publications, and if librarian involvement demonstrates a trend with citation counts. **Methods:** A search in OpenAlex was conducted to identify English-language systematic and scoping reviews published by University-affiliated authors. No publication date filter was used. Data extraction was completed by the authors. **Results:** Sixty-eight KS publications between 2007 - 2025 were found. Thirty-eight publications included librarian involvement, 8 of which were UPEI librarians. The Faculty of Science engaged librarians the most. The highest average citation counts occurred on KS with librarians as consultants (n=320 citations). A negative binomial regression model revealed inconclusive results for the individual effects of librarian involvement and open licensing on citation counts. However, their combination showed a statistically significant synergistic effect, with co-occurrence of both factors associated with substantially increased citations. **Discussion:** UPEI authors have been conducting KS projects since 2007 and while the majority of KS included librarians, only 21% were UPEI librarians, indicating that librarians at partner institutions are essential in supporting UPEI authors. Overall, librarian involvement was represented mostly as coauthors but interestingly, publications with librarians as consultants had the highest average number of citations even though it only represented 6% of the KS. An identified area of opportunity is Atlantic Veterinary College, where only 28% of KS publications include librarian involvement.

# Abstract: CP20. Exploring ChatGPT's potential in systematic review search strategies

Amy Kamel^1^, Catherine Boden^1^, Janice Kung^2^

^1^University of Alberta, ^2^University of Saskatchewan

**Introduction:** The adoption of generative AI (genAI) tools like ChatGPT present opportunities for enhancing efficiency through automation. While genAI use is increasingly commonplace in academia and research, the capabilities and limitations of such tools to support systematic review (SR) searches has yet to be explored. **Methods:** We recruited Canadian health librarians to participate in a two-stage crossover study followed by interviews to elicit librarian perceptions of using ChatGPT for SR searching. Each librarian developed two Ovid MEDLINE searches, one using ChatGPT and one without. ChatGPT training was provided to participants when they were required to use ChatGPT. Interview transcripts were coded for themes using qualitative content analysis by three independent coders. **Results:** Eight of the ten participants preferred traditional searching methods over the use of ChatGPT. Themes that emerged through inductive coding of the transcripts include: Librarian Preferences, Barriers to Adoption and Risks, Value and Strengths of ChatGPT, Adoption Bridging, and Positioning AI within Librarian Practice. **Discussion:** This study provides practical examples of how health librarians may use ChatGPT to expedite their search development process. It also revealed the potential and key limitations of ChatGPT use in its current form. This study highlights the importance of staying informed on the development of GenAI tools.

# Abstract: CP21. Evolving our methods through a test and learn program: implementing artificial intelligence in information retrieval for health technology assessment

Robin Featherstone, Matthew Morris, Danielle MacDougall, Sharon Bailey, David Kaunelis

Canada's Drug Agency

**Introduction:** Research Information Services at Canada's Drug Agency has adopted a test-and-learn program to evaluate the performance of automation or Artificial Intelligence (AI) tools for informational retrieval tasks in health technology assessment. **Methods:** We identified 51 promising tools using a targeted internet search, evaluated these tools with standardized criteria, tested 3 highly ranked tools against usual practice search approaches, developed implementation recommendations, and initiated a pilot to build custom AI tools for targeted search tasks with the potential for large efficiency gains. **Results:** Our program built an evaluation instrument for automated or AI search tools for use by any evidence synthesis producers. Comparative tool rankings allowed us to identify promising tools for further testing against usual practice. From our testing, we learned that available tools had low sensitivity for database searching but could assist with specific tasks (e.g., keyword generation for strategy development) for modest efficiency gains. **Discussion:** Our ongoing work builds on these findings to target AI app use for grey literature searching where we anticipate larger efficiency gains than with database searching. We are building custom AI tools to implement recommendations and to semiautomate established search approaches using checklists for grey literature.

# Abstract: CP22. A longitudinal study of subject headings created by generative AI models for use in evidence synthesis

Mê-Linh Lê, Christine Neilson, Caroline Monnin

University of Manitoba

**Introduction:** Artificial intelligence is impacting nearly every stage of the evidence synthesis process. Generating appropriate subject headings for a literature search is an area where GenAI models often fall short, but these tools are constantly changing. This longitudinal study examines GenAI models’ performance when suggesting subject terms for comprehensive search strategies. **Methods:** We are testing free and paid versions of ChatGPT, Gemini, and Claude using prompts designed to generate MeSH, EMTREE, CINAHL, and APA PsycINFO subject terms. Judgement sampling was used to gather published reviews and related search histories from a variety of health sciences topics. Included reviews were co-authored by a librarian, used a peer-reviewed search, provided at least a MEDLINE search, and were only accessible behind a paywall. An additional original research question and peer reviewed search was devised by the authors. These searches act as gold standard comparators for the subject terms generated by the models. Prompts are run every two weeks. The alignment of GenAI model-suggested subject terms with the corresponding gold standard searches, and the occurrence of hallucinations will be analysed. **Results:** Preliminary results will be available in mid-2026. **Discussion:** Our results will provide the first longitudinal analysis on the performance of large-language models to suggest subject terms for evidence synthesis research. These results will help search experts and researchers to make informed decisions on whether to integrate GenAI tools into their search strategy development process.

# Abstract: CP23. Assessing the performance of the National Library of Medicine's Medical Text Indexer - NeXt-Generation (MTIX) MEDLINE indexing algorithm

Alexandre Amar-Zifkin^1^, Rosie Le Faive^2^, Emma Garlock^3^, Taline Ekmekjian^4^, Virginie Paquet^1^, Tara Landry^1^

^1^Université de Montréal, ^2^University of Prince Edward Island, ^3^University of Ottawa, ^4^Public Health Agency of Canada - Agence de la santé publique du Canada

**Introduction:** Since April 2022, indexing of MEDLINE records is performed predominantly by algorithm, with occasional human intervention. Recent research has raised concerns about indexing algorithms’ capacity to accurately identify meaningful elements of publications. This identification is a necessary precondition to communicating them to searchers through indexing. Concerns have included the risk of the algorithm overlooking one or more relevant concepts, not making use of appropriately precise controlled vocabulary, or assigning incorrect terms due to rhetorical or technical language. The indexing algorithm from 2022-2024 (MTIA) was a rule-based system with exceptions added over time; a machine learning model (MTIX) was implemented in 2024. NLM Technical Bulletins have noted general improvements in MTIX compared to MTIA. Recent research has explored particularities of evocative language and found improvements in discerning human from nonhuman-animal subjects. **Methods:** This work will replicate and improve on a previous project that assessed overall MTIA indexing performance. Our study will assess MTIX performance by reviewing a large, recent and random sample of MTIX-indexed records to determine whether their main concepts are adequately represented. Using a web-form displaying the journal, title and abstract of a record, our screeners will identify key concepts that, per their searching experience, would be used to retrieve it and similar records. After establishing agreement between screeners, we will compare the consensus-concepts to the indexing applied by MTIX. We will compare these findings to previous research, noting emergent trends or issues. **Results & Conclusion:** We intend to make our web-form freely available online. Other results are forthcoming.

# Abstract: CP24. Impact of the College of Physicians and Surgeons of British Columbia Library closure: a qualitative study

Colleen Pawliuk^1^, Shannon Cheng^2^, Edlyn Lim^3^, Asli Ozer^4^, Hal Siden^1^

^1^Department of Pediatrics, BC Children's Hospital, ^2^Provincial Health Services Authority, ^3^University of British Columbia, ^4^BC Children's Hospital Research Institute

**Introduction:** The College of Physicians and Surgeons of British Columbia (CPSBC) announced the closure of their library in March 2024, leaving some physicians without library services due to a lack of academic or health authority affiliation. The CPSBC Library supported all registered physicians in BC with traditional library services, such as self-service resources, mediated literature searches and full-text article retrieval. **Objective(s):** To understand how the closure of the CPSBC Library affected the work of physicians in BC. **Methods:** Using a qualitative descriptive design methodology, we will conduct semi-structured interviews with 15-20 physicians about their experience using the CPSBC Library and of its closure. All interviews will be transcribed and then analyzed using deductive and inductive thematic analysis. **Results:** We have currently completed interviews with 17 physicians and will share the results of our analysis.

# Abstract: CP25. Améliorer la découvrabilité de la littérature grise en français d'un dépôt institutionnel : le cas PhareSST

Catherine Dufresne, Samuel Hébert, Bénédicte Nauche

Institut de recherche Robert-Sauvé en santé et en sécurité du travail

**Introduction:** L’IRSST est un centre de recherche spécialisé en santé et sécurité du travail (SST) qui publie ses propres rapports, guides et fiches. Majoritairement francophone, cette littérature grise vise scientifiques, intervenants en SST mais aussi travailleurs. De plus, l’IRSST souhaite jouer un rôle influent hors du Québec. Dans ce contexte, optimiser la découvrabilité du contenu de son dépôt institutionnel, PhareSST, est incontournable. **Description:** Une étude des moyens proposées pour améliorer la découvrabilité de contenus non-anglophones été faite dès la phase d’implantation de PhareSST. Une liste d’actions à entreprendre a été dressée et instaurée au premier instant de la mise en marche du projet, par exemple la traduction des mots-clés utilisés. Mais quelques obstacles, entre autres d’ordre financier, ont forcé la révision de la stratégie. **Résultats:** Une grille d’analyse a été utilisée afin de voir si les actions prises étaient conformes aux principes FAIR. De plus, une analyse de l’origine des téléchargements sur PhareSST est en cours. Cependant, la question demeure: comment mesurer objectivement et efficacement l’impact des moyens utilisés afin d’augmenter la découvrabilité de notre contenu francophone? **Discussion:** En tant qu’institut de recherche en SST, l’IRSST tient à faire rayonner la connaissance qu’elle produit. Pour se démarquer, une panoplie d’actions doivent encore être déployées, malgré les limites financières et humaines.

# Abstract: CP26. What is an academic library website for? Administrative reflections across academic and health sciences contexts

Susanna Galbraith, David Kemper

McMaster University Libraries

**Introduction:** Academic library websites are critical digital interfaces for service delivery, resource discovery, and institutional representation. Yet their strategic purpose is often ambiguously defined, especially in complex, evolving contexts. While literature highlights user-centered design and web governance, few studies examine how library administrators conceptualize the website’s role in relation to institutional missions. This study investigates how library leaders at a large Canadian university with a strong health sciences mandate articulate the purpose of academic library websites, and how these views shape governance and design decisions. **Description:** Approved by the university's research ethics board, the study used semi-structured interviews with associate university librarians and directors overseeing operations, digital strategy, and health sciences services. Conducted over three months in 2025 via video conferencing, interviews were transcribed and thematically analyzed. The protocol explored website functions, content priorities, institutional context, governance models, and stakeholder alignment. Surveys and focus groups were considered but set aside in favor of interviews for their depth and nuance. **Outcomes:** Preliminary themes include access and discovery, space and service information, strategic positioning, outreach, subject-specific differentiation, and the website as a navigation hub. Analysis is ongoing and finalized themes will be presented as available. **Discussion:** Findings have informed internal discussions on content strategy and design priorities. Strengths include depth of inquiry and relevance to planning; limitations include a single institution focus and absence of user perspectives. Future directions may include user research and tools to support strategic decision-making. The presentation will conclude with reflections on the evolving role of academic library websites.

# Abstract: CP27. Advocating through headlines: collaborating with the media to champion medical libraries and library staff

Amanda Ross-White, Kaitryn Campbell

Queen's University

Collaboration with the media can be an effective advocacy strategy to promote the value of and protect medical libraries and their staff. This case study examines how public storytelling became a vital tool in addressing job cuts, raising awareness of medical libraries, and influencing institutional decision-making. Following the elimination of two positions and closure of the library at St. Joseph's Healthcare Hamilton (SJHH) in early 2024, a collective response was launched by affected staff and allies. This included strategic engagement with local journalists, letters to hospital leadership, and social media amplification. The resulting media coverage provided a platform to communicate the essential role of medical libraries in evidence-based care. This presentation reviews the methods used to frame the narrative, engage diverse stakeholders, and sustain public interest. The media coverage prompted a response from hospital executives. Notably, leadership agreed to facilitate an external review to help ensure library services were in-line with current standards. The review resulted in the reinstatement of many library services and new staff hiring. The campaign also prompted renewed discussions around the visibility and value of medical librarianship, particularly in the face of increasing fiscal constraints and hospital workforce restructuring. This case demonstrates that medical librarians can and should play an active role in shaping public discourse about their profession. Where libraries are at risk of being undervalued, strategic storytelling can be a powerful tool to protect the profession and inform broader conversations about evidence-based care.

# Abstract: CP28. Bridging academic and clinical practice: building partnerships between the Bikram S. Dhillon and Family Medical Library and the William Osler Health System Library

Janice Thompson^1^, Victoria James^2^, Melissa Helwig^2^

^1^William Osler Health System Library, ^2^Toronto Metropolitan University

Hands-on clinical experience is a cornerstone of medical education. Residents spend much of their training in hospitals and clinics, while medical students look forward to clerkship for their supervised, practice-based learning. As Toronto Metropolitan University's (TMU) School of Medicine (SoM) prepared to open, building relationships with the local healthcare institutions was essential. Among these, the collaboration between the Bikram S. Dhillon and Family Medical Library and the William Osler Health System Library has become central to supporting learners across academic and clinical contexts. As our healthcare and academic landscapes prepared to welcome learners, it was important for our health libraries to foster meaningful collaborations that enhance evidence-based practice, research, and education. Fostering a connection between our two library systems allowed us to leverage the skills and services at both institutions to support the new medical school. Together, we are able to explore and co-develop initiatives such as shared education programs, referral processes, subject-guide content, and collaborative support services that benefit students, clinicians, and researchers alike. This presentation explores how partnerships between academic and clinical libraries can bridge institutional silos, foster knowledge exchange, and support the continuum of learning and care. Participants will gain insights into building sustainable partnerships that leverage the strengths of both academic and clinical settings. Emphasis will be placed on communication, mutual goals, and the importance of cultivating trust and shared leadership. By the end of the session, participants will be able to identify strategies for initiating and sustaining library partnerships across academic and clinical contexts.

# Abstract: CP29. Mind the gap! Adding a clinical librarian to neurology rounds to address knowledge gaps

Elizabeth Frakes, Shawn Steidinger

University of Utah

**Introduction:** Studies have shown that as many as 15-20 questions per day can go unanswered as a clinician is seeing patients either due to lack of time or expertise in finding literature. The clinical librarians at the Eccles Health Sciences Library at the University of Utah aimed to address this issue by partnering with the Neurology Consult Service to join clinical rounds in order to provide evidence for any clinical questions that arise around patient care, diagnosis, treatment, outcomes, and standards. **Description:** The clinical librarians join table rounds as well as bedside rounds 2-3 times per week in the main hospital, rehabilitation hospital, and cancer hospital. They send an email with search strategies, a summary of the evidence, and relevant full text articles to the entire rounding team consisting of attendings, residents, and medical students. **Results/Outcomes:** The librarians saw immediate success with over 150 questions answered during the first year of the service and created a searchable repository in a secured shared site so dissemination can reach the entire Neurology department. Clinical staff have added quotes and citations directly into the patient chart notes. Librarians analyzed the clinical questions for themes and duplicates in order to uncover knowledge gaps. **Discussion:** Feedback has been overwhelmingly positive, and the responses have helped fulfill the institution's mission of advancing knowledge and well-being. The librarians were invited to present at Neurology Grand Rounds on the knowledge gap analysis as well as the overall rounding experience. The librarians hope the analysis can lead to changes in the curriculum.

# Abstract: CP30. Listening to students: a UX approach to nursing subject guide redesign

Jody Nelson, Ali Foster, Danielle Deschamps

MacEwan University

**Introduction:** Of the many Springshare-powered subject guides at our undergraduate, teaching-focused university, the Nursing Guide ranks as the most used, with over 30,000 annual visits, yet it has not undergone any significant revision in over 10 years. To that end, the Nursing librarian team, which includes our User Experience Librarian, are undertaking a mixed-methods, participatory research approach to redesigning the Nursing Guide that centres the Nursing student experience. The primary research questions guiding our study: 1. When and why is the guide being accessed, for which contexts and purposes, and by whom? 2. How are students navigating and engaging with the guide? What are their challenges, blocks, unmet needs, or undiscovered gaps? What do students want from the Nursing Guide? **Methods:** We are using a mixed-methods approach to data collection: Phase 1: Quantitative data will be collected via a survey sent to all Nursing students. It will collect demographic information, such as year of study and current courses, as well as questions related to the use and relevance of the Nursing Subject Guide. Phase 2: Applying UX principles through a participatory research lens, we will conduct think-alouds to collect rich qualitative data on how students navigate the guide in practice. **Results:** We will share the results of our quantitative data analysis, as well as our experiences, observations, and preliminary insights from the student think-alouds. Participants will have the opportunity to learn about our student-engaged UX approach and how this can be applied to their own quality improvement initiatives for library users.

# Abstract: CP31. Supporting great teaching: guidance for accessible, engaging, and effective hospital library instruction

Raluca Serban, Ashley Farrell, Melanie Anderson

University Health Network

**Introduction:** We are a team of library professionals dedicated to enhancing the instructional processes within our team while also designing accessible and impactful learning experiences for our users. Based on a literature search of both published and grey literature related to accessible instruction and best practices, we found no resource that addressed best practices for instruction in hospital libraries. **Description:** We developed a comprehensive guide for our library team covering internal library processes, accessibility, multimodality, interactivity, and best practices for diverse learners. We will share our process for developing the guide, as well as the guide itself. **Outcomes:** It was important to assess the usability, accuracy, and completeness of the guide, to ensure that our team could easily develop and offer the most accessible instruction possible. Feedback will be presented from several sources: test users applying the guide to sample instruction products, our institution's Accessibility Council, and surveys of our team as well as outside health sciences library professionals. **Discussion:** While accessibility remains a guiding principle, it comes with real world constraints such as institutional, technological, or contextual challenges. Our guide offers practical strategies for enhancing the planning and delivery of asynchronous and synchronous sessions. The guide will officially launch in January 2026, followed by broad sharing across health sciences libraries and an anonymous usage poll in summer 2026. A regular review schedule will help keep the guide relevant, inclusive, and responsive to evolving needs.

# Abstract: CP32. Using the 'train the trainer' approach to teach information literacy to medical learners

Jackie Phinney^1^, Angela Hogan^2^

^1^Dalhousie University Libraries, ^2^Dalhousie Medicine New Brunswick

**Introduction:** Information literacy instruction is vital for ensuring that medical learners can effectively incorporate evidence into clinical practice. However, efforts to instruct this group can be hindered by curriculum schedules, and clinical supervisors' information practices can influence learners in the real-world environment. **Description:** To strengthen learners' information skills in the clinic, a continuing professional development (CPD) course titled 'Bringing Information Literacy into your Clinical Teaching' was developed for medical school faculty. Guided by adult learning theory and principles of instructional design, this asynchronous course explores facets of information literacy and evidence-based practice in the modern clinical setting, while incorporating teaching pearls to help clinical educators have conversations about these topics with medical learners. By integrating the perspectives of an experienced CPD educator and physician colleagues, this course was also designed to meet professional accreditation standards. **Outcomes:** The course launched in the winter of 2026, and while it is still gaining momentum this presentation will provide an in-depth overview of the course's conceptualization, its design elements, and its content. It will also discuss lessons learned when expanding one-shot content for wider use, finding confidence when teaching in new formats, and the importance of support networks when evolving as an educator. **Discussion:** Libraries are key partners in supporting learner growth in the clinical environment. By using a 'train the trainer' approach to teach information literacy in a medical school setting, library instruction can continue shaping learner practices for years to come.

# Abstract: CP33. Is this still, ok? The case of the skeleton in the library

Raluca Serban, Jessica Babineau

University Health Network

**Introduction:** What began as a straightforward question: 'Should this skeleton still be on display in the library?' evolved into a deeper inquiry. This presentation will explore a hospital library’s investigation into the origins, ethics, and future of human remains held in our library space. It will also discuss the experience of collaborating with an academic program to create a meaningful, ‘real-world’ learning experience for students in a forensic anthropology program. **Description:** As part of this project, a literature review was first conducted to explore the ethics of the use and display of skeletal human remains in medical education. This exploration led to concerns and questions about the origins, educational value, care, and dignity of the individual in our library. Our team engaged experts from our organization's bioethics department and an academic forensic anthropology department to assess the skeleton’s provenance and determine actionable steps to move forward. **Outcomes:** Through collaboration with bioethics and forensic anthropology, this problem was leveraged into a learning opportunity for budding forensic anthropologists. Through this exercise, our team received a report confirming the individual’s descent, age and sex. Further, practical recommendations were provided by a forensic anthropology expert, informing our team’s decisions. **Discussion:** Throughout this project, we learned about institutional accountability, the ethics of human artifact stewardship, and the importance of respectful decision-making when the past meets the present. Through this presentation, we will share our process, findings, actions, and lessons learned.

# Abstract: CP34. Embedded outreach: clinical librarians and the electronic health record

Elizabeth Frakes, Shawn Steidinger

University of Utah

**Introduction:** This paper describes the development process, implementation steps, service structure, and outcomes of the library's evidence request service embedded in the electronic health record (EHR). **Methods/Description:** At the Eccles Health Sciences Library, clinical librarians have embedded their Expert Evidence Consult Service (EECS) into the hospital's EPIC system using the existing messaging feature. Librarians worked with Epic analysts to develop an alert that generates an email notifying the team of a submission. Clinical librarians with EHR access (granted after EPIC training and permission from system administrators) can log in to view the message which includes clinical question, patient record number, and priority level. This EHR access permits the librarians to view the relevant patient record, enabling a patient-specific tailored response that includes a summary of the literature, attached articles, and/or links to collections of PubMed citations. **Results:** The EECS debuted in EPIC in Fall of 2020. Statistics collected include how many questions answered, repeat users, and department. Various marketing pushes have been implemented to varying success. A feedback survey was first implemented in RedCap in the Fall of 2024. Questions ask about satisfaction of the material, timeliness, impact on practice, and the likelihood to use the EECS in the future. **Discussion:** Feedback from the survey has been uniformly positive. Satisfaction with the timeliness, accuracy, and relevance of the librarian-provided evidence is also demonstrated by the existence of repeat users. Outreach strategies include invited presentations to c-suite meetings, classes, orientations and clinical faculty meetings, and a proposed marketing push with a polished service flyer.

# Abstract: CP35. From insight to action: elevating library visibility through targeted outreach

Janice Thompson, Melissa Paladines

William Osler Health System

**Introduction:** Following a comprehensive survey of library services, our team identified key service gaps and launched a strategic outreach initiative. This initiative evolved into an annual promotional campaign aimed at increasing awareness and engagement with library services and resources across the organization. **Description:** Using design tools such as Canva, we developed visually engaging promotional materials—including email banners, flyers, posters, and newsletters— to spotlight underutilized resources and core services. To ensure relevance and maximize impact, we adopted a targeted outreach approach by tailoring content to specific healthcare specialties. This customization allowed us to speak directly to the unique needs of various departments, fostering stronger connections and encouraging resource utilization. **Outcomes:** The campaign not only improved visibility of library offerings but also positioned the library as a proactive and collaborative partner within the organization. Through consistent and focused promotional efforts, we cultivated relationships with clinical and administrative teams, leading to new opportunities for cross-departmental collaboration. **Discussion:** This presentation will share practical strategies for designing and implementing targeted outreach, highlight examples of promotional materials, and discuss lessons learned in building sustainable partnerships. Attendees will gain insights into how strategic communication and visual design can elevate the role of the library.

# Abstract: CP36. Initiatives bibliothérapeutiques à l'hôpital-Hospital bibliotherapeutic initiatives

Alix Pincivy^1^, Mylène Lalonde^2^

^1^CHU Sainte-Justine, ^2^ETS

**Introduction :** La bibliothèque de l’hôpital Sainte-Justine a mis en place deux initiatives présentant la lecture comme un moyen, pour les patients et les familles, de s’outiller pour faire face à leur problématique, ou de se changer les idées et se relaxer. **Description :** En collaboration avec les intervenant-e-s des cliniques, des affiches « Des lectures qui font du bien » ont été créées pour les salles d’attentes afin de permettre de faire les liens entre les collections de la bibliothèque et les patients/famille. L’équipe soignante du centre intégré pour les troubles de la conduite alimentaire (CITCA) et la bibliothécaire ont mis en place des ateliers bibliocréatifs hebdomadaires destinés aux patient-e-s hospitalisé-e-s. **Résultats/Discussion :** Cette présentation vous présentera les processus d’implantation des initiatives et de partenariat avec le personnel soignant ainsi que le retour d’expérience.

# Abstract: CP37. Mapping the bioinformatics resource landscape: an analysis of LibGuides across Canadian universities

Grailing Anthonisen^1^, Lisa Banks^2^

^1^McGill University, ^2^New Brunswick Public Libraries

The introduction of LibGuides created a seismic shift in bioinformatic information dissemination. This study explores approaches to resource selection and subject guide development by reviewing bioinformatics LibGuides across Canadian academic libraries. Our project aimed to determine a consistent overlap of frequently recommended materials that would spell out a canon of bioinformatics resources. We undertook a manual review of the 11 bioinformatics-specific LibGuides. We found four LibGuides focused solely on bioinformatics and seven guides with subsections dedicated to bioinformatics. Overall, there were 566 resources distributed across the guides, with little overlap across guides. Most (n=440) were distinct resources. The most common resource type was database and the resources that appeared the most were BLAST Database, Protein Data Bank, and PubMed, which appeared five times. We identified clear variations in the intended audience for these subject guides, as well as commonalities across all 11. The diversity of listed resources reflects the diversity and interdisciplinary nature of bioinformatics. However, despite this variety, there was still a trend in those resources' funding or hosting. They are often provided by American institutions, via American funding. This raises concerns for information professionals when it comes to access to and veracity of data to use and teach bioinformatics resources, highlighting the pressing need for strategies that advocate for more diverse resource selections and cultivate relationships with international data repositories.

# Abstract: CP38. Guiding AI principles and curriculum: a library-led approach at the Canadian College of Naturopathic Medicine (CCNM)

Melissa Caines

Canadian College of Naturopathic Medicine (CCNM)

Generative AI (GenAI) is reshaping academic health education and increasing expectations for institutional response. At the Canadian College of Naturopathic Medicine (CCNM), the library partnered with campus colleagues and an external GenAI consultant to develop institutional GenAI principles and faculty and student guidance, creating a sustained entry point for embedded librarianship. **Introduction:** As GenAI use accelerates, health programs are navigating how to encourage innovation while protecting academic integrity and patient privacy. GenAI principles and user-facing guidance reduce uncertainty and support responsible practice. At CCNM, this work helped shift library teaching from primarily request-based sessions toward embedded curricular support. **Description:** As Chair of CCNM’s AI Task Force, Melissa collaborates with interdepartmental partners across CCNM and an external GenAI consultant. Using CCNM community input and national frameworks, including U15 and Government of Canada resources, the group developed GenAI principles and practical guides for responsible, context-appropriate GenAI use. **Outcomes:** This work increased the visibility of the CCNM Library. Embedded teaching now covers academic integrity, digital media literacy, GenAI and patient privacy, search strategies, and research skills for clinical questions and systematic reviews, with additional curriculum development in progress. **Discussion:** This initiative demonstrates how an academic health sciences library uses strengths in information and GenAI literacy to support CCNM's GenAI response and establish embedded librarianship at CCNM. Collaborative development of principles and practical guidance, informed by national frameworks and CCNM community input, supports responsible practice and creates durable opportunities for curriculum-integrated library teaching.

# Abstract: CP39. Leveraging foundational information literacy practices and cutting-edge technology to prepare the next generation of healthcare professionals

Laurel Scheinfeld, Sunny Chung, Jessica Koos

Stony Brook University Health Sciences Library

**Introduction:** This presentation details an assessment of SEARCH AI, a home grown semantic search tool built using generative AI that empowers patrons to query the library catalog using plain, natural language. **Methods:** Three health sciences librarians conducted a comparative study to assess the efficacy of SEARCH AI against traditional catalog searching. The study utilized actual Fall 2025 undergraduate health sciences student research topics to query the catalog. For each topic, a traditional search was compared to an AI-generated search. Data collected included the number of results, and the relevancy of the first ten results to the student's research need. Furthermore, the AI- generated Boolean search strings were assessed for accuracy using a subset of criteria from the PRESS checklist. **Results:** Initial findings suggest SEARCH AI has the potential to enhance the discoverability of resources for novice users, transforming conversational searches into more complex Boolean queries. **Discussion:** The results will be used to directly inform the development of an integrated library instruction module focused on educating health sciences students on the strategic, critical use of this AI search feature, teaching them not only how to use the tool but also how to interpret its output (specifically the generated Boolean strings) to foster advanced search proficiency - a cornerstone of evidence-based practice.

# Abstract: CP40. Loon lens: future in focus - building search strategies with AI assistance

Yuhong Yuan^1,2,3^, Ghayath Janoudi^4^, Mara Rada^4^

^1^Department of Medicine, Western University, London, Ontario; ^2^London Health Science Centre, London, Ontario; ^3^Department of Medicine, McMaster University, Hamilton, Ontario; ^4^Loon Inc.

**Background:** GenAI-assisted search strategies are gaining momentum. Loon Lens is a Canadian initiative and the first- ever scientifically validated agentic AI platform for systematic literature reviews. Loon Lens supports key steps in evidence synthesis, including developing eligibility criteria, building search strategies, running literature searches within OpenAlex, and conducting title, abstract, and full-text screening, as well as data extraction. It is HTA-compliant, citable, and demonstrates high performance. **Methods:** We tested Loon Lens as an independent comparator to generate multiple literature search strategies based on predefined inclusion criteria, and we compared its performance with that of an experienced information specialist and researcher. With humans kept in the loop, agentic AI is not intended to replace human expertise but has been shown to enhance efficiency in literature searching. **Results and discussion:** We will share our experience using Loon Lens to refine literature search strategies and reduce human time investment in the systematic review process. We will also discuss the strengths and limitations of AI tools in literature searching. As a research assistant and valuable collaborator, Loon Lens—when used alongside librarians—has the potential to substantially strengthen the quality, consistency, and timeliness of future systematic review workflows.
